# Differential methylation of the type 2 diabetes susceptibility locus *KCNQ1* is associated with insulin sensitivity and is predicted by CpG site specific genetic variation

**DOI:** 10.1016/j.diabres.2019.01.008

**Published:** 2019-02

**Authors:** Ushma J. Shah, Weijia Xie, Allan Flyvbjerg, John J. Nolan, Kurt Højlund, Mark Walker, Caroline L. Relton, Hannah R. Elliott, for the RISC consortium

**Affiliations:** a Institute of Genetic Medicine, Newcastle University, Newcastle upon Tyne NE1 7RU, UK; b MedGenome Labs Ltd., Bangalore 560 099, India; c Peninsula School of Medicine and Dentistry, Exeter EX2 5DW, UK; d Steno Diabetes Center Copenhagen, The Capital Region of Denmark and University of Copenhagen, Copenhagen, Denmark; e European Association for the Study of Diabetes (EASD), 40591 Düsseldorf, Germany; f Steno Diabetes Center Odense, Odense University Hospital, DK-5000, Denmark; g Institute of Cellular Medicine, Newcastle University, Newcastle upon Tyne NE2 4HH, UK; h MRC Integrative Epidemiology Unit at the University of Bristol, Bristol BS8 2BN, UK; i Population Health Sciences, Bristol Medical School, University of Bristol, Bristol BS8 2BN, UK

**Keywords:** Insulin sensitivity, *KCNQ1*, Methylation, SNP, Type 2 diabetes

## Abstract

**Aims:**

Epigenetic mechanisms regulate gene expression and may influence the pathogenesis of type 2 diabetes through the loss of insulin sensitivity. The aims of this study were to measure variation in DNA methylation at the type 2 diabetes locus *KCNQ1* and assess its relationship with metabolic measures and with genotype.

**Methods:**

DNA methylation from whole blood DNA was quantified using pyrosequencing at 5 CpG sites at the *KCNQ1* locus in 510 individuals without diabetes from the ‘Relationship between Insulin Sensitivity and Cardiovascular disease’ (RISC) cohort. Genotype data was analysed at the same locus in 1119 individuals in the same cohort. Insulin sensitivity was assessed by euglycaemic-hyperinsulinaemic clamp.

**Results:**

DNA methylation at the *KCNQ1* locus was inversely associated with insulin sensitivity and serum adiponectin. This association was driven by a methylation-altering Single Nucleotide Polymorphism (SNP) (rs231840) which ablated a methylation site and reduced methylation levels. A second SNP (rs231357), in weak Linkage Disequilibrium (LD) with rs231840, was also associated with insulin sensitivity and DNA methylation. These SNPs have not been previously reported to be associated with type 2 diabetes risk or insulin sensitivity.

**Conclusion:**

Evidence indicates that genetic and epigenetic determinants at the *KCNQ1* locus influence insulin sensitivity.

## 1 Introduction

Decreased insulin sensitivity is associated with a wide range of common disorders including type 2 diabetes, obesity, hypertension and cardiovascular disease. Genome-wide association studies (GWAS) have identified over 100 type 2 diabetes susceptibility loci [1]. However, many appear to impact upon β-cell function and insulin secretion rather than insulin sensitivity [1], [2] leading to the potential importance of non-genetic factors such as epigenetic modifications.

Epigenetic variation has recently become the focus of considerable interest in the domain of common complex diseases, due to the role of epigenetic mechanisms in gene regulation. DNA methylation is most commonly studied, largely due to its covalent stability and ease of measurement. A detailed overview of the current literature linking type 2 diabetes and DNA methylation has been recently published [3]. DNA methylation has been associated with type 2 diabetes in a number of case-control studies, conducted on both genome wide and locus specific methylation data in various tissues [4], [5], [6], [7]. DNA methylation has also been identified as a predictor of incident disease [8]. In addition to its association with type 2 diabetes, evidence also suggests DNA methylation is associated with insulin sensitivity measured by HOMA-IR [9], [10].

In this study we explore locus-specific DNA methylation patterns in the gene *KCNQ1,* which has established links to type 2 diabetes aetiology [7]. A number of SNPs at the 11p15 locus (which includes *KCNQ1*) have recently been implicated in type 2 diabetes in both European and Asian populations [1], [11]. DNA methylation in a differentially methylated region ∼769 kb distal to *KCNQ1* has previously been associated with the genotype of one these SNPs, rs2334499 [12], suggesting that methylation at the 11p15 locus could be important in the aetiology of type 2 diabetes or its associated traits. We targeted an intronic region within the *KCNQ1* gene itself, as limited previous evidence suggested that inter-individual variation in DNA methylation existed in this region [13].

The primary aim was to assess the relationship between DNA methylation at the *KCNQ1* locus and measures of insulin sensitivity and β-cell function in a cohort of healthy individuals without diabetes. Individuals without diabetes were studied in order to remove the potentially confounding effects of the disease state (e.g. hyperglycaemia and dyslipidaemia) on the measures of interest. Here we describe the DNA methylation-phenotype relationship at this locus and consider the role of the underlying genetic architecture on the determination of DNA methylation and in turn the potential influence of these factors on phenotype in this healthy population.

## 2 Subjects, material and methods

### 2.1 Study cohort

Samples were derived from the *Relationship between Insulin Sensitivity and Cardiovascular disease* (RISC) cohort. This is a collection of individuals aged between 30 and 60 years recruited from 19 centres in 14 countries across Europe. The recruitment methods of the study have been described previously [14].

Data collected as part of this study were generated between September 2010 and June 2011 at Newcastle University, UK.

DNA was extracted from whole blood using a Nucleon BACC2 kit (Tepnel Life Sciences, UK) according to manufacturer’s instructions. All individuals with DNA samples who had undertaken metabolic analysis were considered for selection (n = 1319). Methylation data were generated in 510 individuals randomly selected from the overall group (n = 1319) who had undertaken a euglycaemic-hyperinsulinaemic clamp and had fasting glucose levels of ≤7 mmol/L measured by clamp or oral glucose tolerance test. 459 of 510 individuals assayed for *KCNQ1* methylation had genotyping, methylation and metabolic data. Further investigation of genotype against insulin sensitivity was conducted in 1119 of 1319 individuals who had both genotype and metabolic data. Additional genotyping of rs231840 was undertaken (see Section 2.4).

Local ethics committee approval was obtained by each recruitment centre (see supplementary appendix). Written consent was obtained from all participants.

### 2.2 Metabolic measures

A number of insulin sensitivity and β-cell function measures were investigated. M value is a measure of insulin sensitivity, and represents the amount of glucose infused during euglycaemic-hyperinsulinaemic clamp per minute per kg of lean body mass, as determined by bioimpedance (Tanita International division, Yiewsley, UK) [14]. Fasting plasma glucose and serum insulin measured were assayed in the fasting stage of an oral glucose tolerance test (OGTT) [14]. Two hour plasma glucose was also assayed during OGTT. Pancreatic β-cell function was assessed by measuring β-cell glucose sensitivity using a model described previously [15], [16]. Homeostasis model assessment-insulin resistance (HOMA-IR) was calculated as (fasting plasma glucose (mmol/L) × fasting serum insulin (mU/L))/22.5. Leptin and adiponectin were measured in fasting serum samples as described previously [17].

### 2.3 Methylation measures

**Fig. 1:**
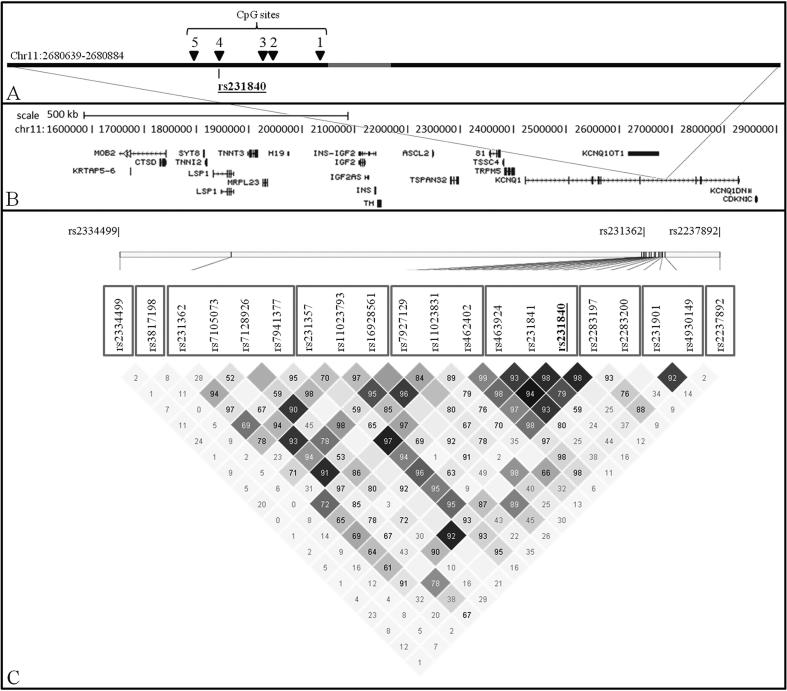
Chromosome 11p15 locus. A: Black bar represents the amplicon generated for Pyrosequencing®. CpG sites analysed are numbered and shown as black triangles. The grey bar represents the location of the Pyrosequencing® primer. Location of rs231840 is shown. B: Topography of region of interest. Gene annotations were taken from the University of California Santa Cruz genome browser (http://genome.ucsc.edu/). C: Linkage disequilibrium plot created from Haploview v4.2. Values indicated are D′ (1 = complete linkage disequilibrium). Colour scheme represents r^2^ values: r^2^ = 0, white; 0 < r^2^ < 1, shades of grey; r^2^ = 1, black. Grey squares around SNP identifiers indicate the blocks as defined by solid spine linkage disequilibrium (SSLD) or singleton SNPs. Type 2 diabetes associated SNPs are shown at top of the panel (rs2334499, rs231362, rs2237892).

Percentage DNA methylation at five CpG sites in the *KCNQ1* locus was determined in a sub-group of 510 RISC individuals using Pyrosequencing**®**. CpG sites were numbered 1–5 and are shown in Fig. 1. PCR primers spanning the 246 base pair region chr11:2680639-2680884 (5′ Biotin-TGG TTA GGA AGG ATA GTT3′ and 5′CAA CCT CAC ATA CTT CTC3′) and a sequencing primer (5′AAT AAA AAC ACA CAA CAA AT3′, chr11:2680741-2680760) were designed using Qiagen PSQ assay design software, v.1.0.6 (Fig. 1).

One µg of genomic DNA was bisulphite modified using an EZ DNA Methylation kit (Zymo Research, Irvine, USA) according to the manufacturer’s instructions. A PCR reaction targeted to the region of interest was then performed containing 40 ng of modified DNA. EpiTECT methylated/unmethylated DNA was used in control reactions (Qiagen, Crawley, UK). In addition to DNA, 2x Q buffer (Qiagen, Crawley, UK), 0.5 µM of each oligonucleotide and HotStar Master Mix (Qiagen, Crawley, UK) were added, to a final volume of 20 µL. Amplification was initiated by denaturation at 95 °C for 15 min, followed by 50 cycles of denaturation at 95 °C for 15 s, annealing at 50.5 °C for 30 s, and extension at 72 °C for 15 s. Finally, there was a further extension at 72 °C for 5 min before the samples were cooled to 4 °C. Five µL of amplicons were utilised for downstream single strand preparation and hybridisation of 0.4 µM sequencing primer using a Qiagen vacuum prep tool and workstation according to manufacturer’s instructions. Pyrosequencing® analysis was conducted on a PyroMark MD system on each sample in duplicate. Assay success rate was 98.2%. The intra-plate co-efficient of variation was 2.2% and inter-plate co-efficient of variation was 3.7%.

### 2.4 Genotyping

One of the CpG sites measured harboured a C > T single nucleotide polymorphism (SNP), rs231840 (CpG 4, see Fig. 1). HapMap data indicated that rs231840 lay towards the 3′ end of a linkage disequilibrium (LD) block spanning approximately 95 kb between chr11:2600454 and chr11:2695235 (NCBI, build 36). SNP data were previously generated on the RISC cohort using an Affymetrix Genome-wide Human SNP Array 6.0 (Affymetrix, Santa Clara, USA). Genotype data from the SNPs lying within this LD block were extracted from the RISC GWAS dataset (a total of 26 SNPs).

In addition, we imputed genotypes for *KCNQ1* SNPs recently found to be associated with type 2 diabetes that were not present on the array, using MaCH. SNPs rs2334499 [12], rs231362 [2] and rs2237892 [18], [19] were imputed in 1004 RISC individuals.

SNP rs231840 is not present on the Affymetrix Genome-wide Human SNP Array 6.0 and could not be imputed from the available data since it was not present in the HapMap reference panel. Genotyping of rs231840 was therefore completed by KBioscience (www.kbioscience.co.uk) using their proprietary KASPar allele specific genotyping technology. 1058 RISC individuals were genotyped successfully (509 from the subgroup of 510 individuals with methylation data and 549 from the remaining cohort of 1319. Duplicates (n = 99) were tested and concordance was 100%. Furthermore, two samples from each genotype were also genotyped using Pyrosequencing®. Methods are available from the authors on request. This quality control established that the KASPar technology was not influenced by methylation which could have biased genotype calls. Concordance between the methods was 100% indicating this was not the case. Genotypes were coded as 0, 1 or 2, reflecting the number of copies of the minor allele individuals had.

### 2.5 Statistical analysis

Linear regression analysis was conducted to investigate the relationship between methylation, metabolic measures and genotype. Outcome variables were normalised using Log_10_ transformation. Where appropriate, adjustment for potential confounders (age, sex, study centre and BMI) was carried out as described in the results. Adjustment for study centre was included to help account for any major population stratification across Europe. Regression analysis outcomes are presented as: (exponentiate of regression coefficient (95% confidence interval), p-value).

Mann-Whitney tests and Fishers exact tests were used to compare sample groups in Table 1.

**Table 1:** RISC cohort characteristics.

Characteristic	Sub-group median (IQR)	Remaining cohort median (IQR)	P value (Mann-Whitney)
N	510	809	
Age (years)	45 (38–51)	43 (36–49)	1.420e−04
Men (%)*	44.9	44.2	0.806
BMI (kg/m^2^)	24.8 (23.0–27.7)	25.1 (22.6–27.8)	0.788
Fasting glucose (mmol/l)†	5.2 (4.8–5.5)	5.0 (4.7–5.4)	1.565e−06
2 h glucose (mmol/l)†	5.7 (4.9–6.7)	5.7 (4.6–6.6)	0.033
Fasting Insulin (mU/L)	4.5 (2.8–6.2)	4.5 (3.0–6.5)	0.895
M value (μmol/min/kg_ffm_)	53.3 (39.6–66.8)	51.8 (38.7–64.7)	0.206
Adiponectin (μg/ml)	7.7 (5.6–10.0)	7.8 (5.5–10.7)	0.388
Leptin (ng/ml)	9.5 (2.7–16.3)	10.0 (4.7–17.9)	0.716
Β-cell glucose sensitivity (pmol/min/m^2^/(mmol/l)	112.6 (76.5–154.8)	113.5 (79.2–162.1)	0.665
HOMA-IR	1.0 (0.6–1.4)	1.0 (0.6–1.5)	0.448

* Fishers exact test.

† Measured by Oral Glucose Tolerance Test. IQR: Inter-quartile range. Sub-group represents samples for which methylation data was generated. Remaining cohort represents remaining samples from the study population (total n = 1319) ffm, fat-free mass.

In total, 30 SNPs were analysed. These comprised of GWAS SNPs, imputed SNPs and genotyped SNP rs231840. SNPs with minor allele frequency of <5% (n = 5) and those which were not in Hardy-Weinberg equilibrium (p < 0.05) (n = 6) were removed from the analysis (combined n = 10).

Where multiple SNPs were analysed, multiple testing was addressed using a Bonferroni correction of unadjusted p-values. The Bonferroni denominator was determined as the sum of the LD blocks plus the number of SNPs not allocated to a block. The *Solid Spine of LD* (SSLD) method as utilised in the program HAPLOVIEW to define blocks. SNPs that had contiguous pairwise D′ values of ≥0.8 were included in a block. The SNPs considered in our analysis formed six blocks with three additional singleton SNPs (Fig. 1). Consequently, the Bonferroni denominator for correction was n = 9.

Where multiple methylation values were analysed, a Bonferroni correction was considered too conservative given the varying degrees of correlation observed between the methylation levels at the 5 CpG sites (rho = 0.23 to 0.89, see results). Hence, given the lack of independence between variables, no correction was applied. Likewise, we did not apply correction to associations tested with metabolic measures due to varying degrees of correlation between them (see Supplementary Table 1).

Statistical analysis was conducted using STATA 11 (STATA corporation, Texas, USA) and R version 3.4.1. The following R packages were used: base, stats, xlsx, Biobase, GEOquery, foreign, meffil.

## 3 Results

### 3.1 Descriptive statistics

Median values for the metabolic parameters listed in the metabolic measures section were within the normal range expected for a population without diabetes. Median values for metabolic measures for both the sub-group (n = 510) and selected cohort (n = 1319) are summarised in Table 1. The sub-group for the methylation study had slightly higher fasting and 2 h glucose in comparison to the rest of the RISC cohort.

Although methylation at rs231840 demonstrated evidence of correlation with average methylation at the other four sites tested (Spearman’s average rho = 0.40, p = 5.16e−07) it was notably weaker than the correlation between the other four sites themselves (Spearman’s average rho = 0.63, p = 1.97e−26). Methylation at rs231840 was therefore analysed separately and average methylation for the other four CpG sites was calculated (Fig. 1, CpG sites 1, 2, 3 and 5).

### 3.2 SNP rs231840 is strongly predictive of rs231840 methylation

Unadjusted regression analysis in the sub-group (n = 510) identified genotype as a strong predictor of methylation at rs231840 (0.447 (0.436, 0.459) p = 2.14e−235).

**Fig. 2:**
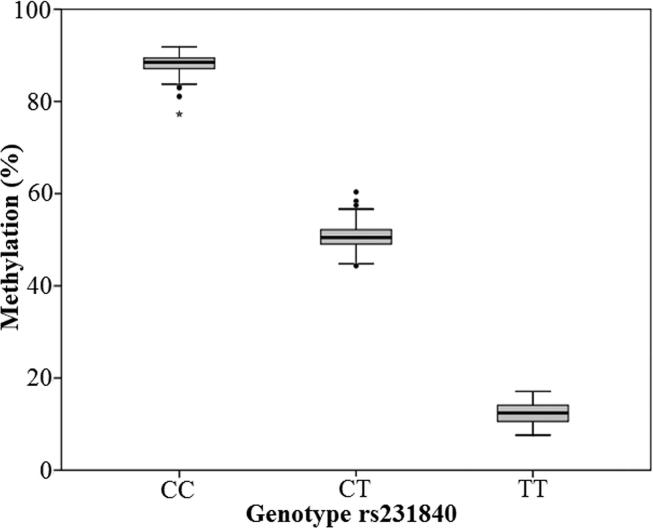
Methylation vs genotype at rs231840. Boxplot showing the relationship between percentage methylation and genotype. Boxes show the median and interquartile range, whiskers represent the minimum and maximum. Closed circles and star are possible outliers.

This is expected, since presence of the minor allele removes the CpG site at rs231840 and therefore also the possibility of methylation occurring. DNA methylation measured at CpG 4 is therefore a proxy for genetic variation at rs231840. A plot of mean methylation grouped by genotype is shown in Fig. 2. The minor allele frequency of rs231840 in the RISC population was 0.30.

### 3.3 Methylation at rs231840 is associated with M-value and serum adiponectin

Regression analysis of rs231840 methylation and metabolic measures (n = 510) showed that methylation predicted M-value (1.002 (1.001, 1.003), p = 0.031) and serum adiponectin (1.002 (1.000, 1.003), p = 0.032). Methylation was not associated with any other metabolic measure. Methylation was not associated with M-value or serum adiponectin in rs231840 C:C carriers. Adjustment for confounders did not alter estimates appreciably.

Regression analysis also showed that methylation at CpG1, 2, 3 or 5 did not predict any metabolic measure tested.

### 3.4 SNP rs231840 in *KCNQ1* is associated with M-value

Genotyping of rs231840 was completed in 1058/1319 RISC individuals (see Section 2.4). Regression analysis of genotype and metabolic measures showed that genotype predicted M (0.95 (0.91, 0.98) p = 0.004). Genotype was not associated with any other metabolic measure (see Supplementary Table 2). Adjustment for confounders attenuated the observed association; M (0.97 (0.94, 1.00), p = 0.076).

### 3.5 SNP rs231840 is associated with local CpG methylation

**Fig. 3:**
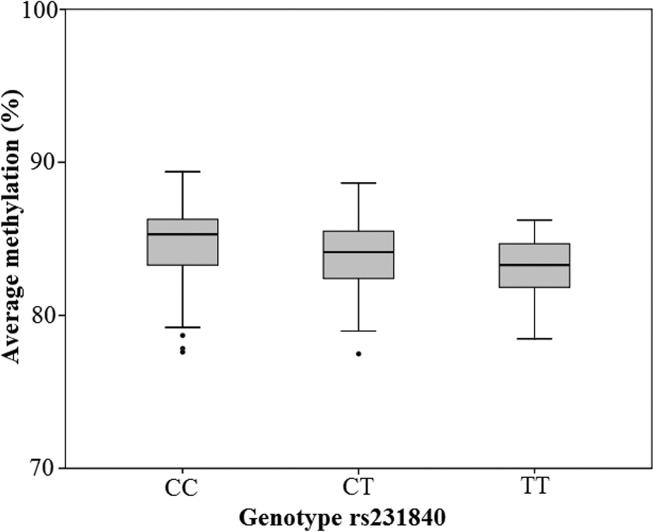
Average methylation at adjacent 4 CpG sites vs genotype at rs231840. Boxplot showing relationship between percentage methylation and genotype. Boxes show the median and interquartile range, whiskers represent the minimum and maximum. Closed circles are possible outliers.

rs231840 genotype adjusted for confounders also predicted average methylation at the local CpG sites analysed, n = 509, (0.990 (0.986, 0.993), p = 1.128e−08). DNA methylation grouped by genotype is shown in Fig. 3. The direct effect of genotype, calculated from r^2^ values, suggested that rs231840 genotype accounted for 6.01% of the variance in methylation at the four local CpG sites. When looking at these CpG sites separately, it was noted that rs231840 elicited the largest effect on CpG 5 (location shown in Fig. 1). Genotype at rs231840 adjusted for confounders predicted methylation at CpG5, (0.970 (0.966, 0.973), p = 4.262e−49). The direct effect on variance was 34.67%. However, local CpG methylation or CpG methylation at any one local CpG site did not robustly predict any other metabolic measures tested.

### 3.6 *Cis KCNQ1* SNPs

An LD plot of SNPs neighbouring rs231840 is shown in Fig. 1. These SNPs were analysed to establish if any other *cis-*SNPs showed evidence of an association with M or adiponectin.

Results of regression analyses are shown in Table 2. SNPs immediately adjacent to rs231840 did not support an association with M-value or serum adiponectin. However, distal SNPs were also associated with these metabolic measures. SNP rs231357 had the strongest association with M-value and serum adiponectin (see Table 2). Analyses at rs231357 adjusted for confounders were still associated after Bonferroni correction, assuming an initial cut-off for association of p < 0.05. The direct effect of genotype at rs231357, calculated from r^2^ values, suggested that rs231357 genotype accounted for 0.57% and 0.58% of the variance in M-value and serum adiponectin respectively. This is compared to the direct effect of genotype at rs231840, which suggested that rs231840 genotype accounted for 0.22% and 0.18% of the variance in M-value and serum adiponectin respectively.

**Table 2:** SNP regression analysis.

			Unadjusted		Adjusted	
Measure	SNP	r^2^ value for LD between SNP and rs231840	Coefficient (95% confidence interval)	p value	Coefficient (95% confidence interval)	p value
M value (μmol/min/kg_ffm_)	rs2334499	0.00	1.02 (0.98, 1.06)	0.341	1.01 (0.98, 1.05)	0.398
	rs3817198	0.00	1.03 (1.00, 1.07)	0.086	1.02 (0.99, 1.06)	0.145
	rs231362	0.18	0.97 (0.93, 1.00)	0.048	0.98 (0.95, 1.01)	0.228
	rs7105073	0.02	0.98 (0.91, 1.05)	0.560	1.01 (0.95, 1.07)	0.694
	rs7128926	0.06	0.99 (0.94, 1.03)	0.504	1.00 (0.96, 1.04)	0.979
	rs7941377	0.03	1.02 (0.95, 1.10)	0.619	1.01 (0.95, 1.08)	0.673
	rs231357	0.41	0.94 (0.91, 0.97)	3.841 e−04	0.96 (0.93, 0.99)	0.004
	rs11023793	0.05	0.98 (0.94, 1.02)	0.317	0.99 (0.95, 1.02)	0.444
	rs16928561	0.02	0.92 (0.86, 0.99)	0.033	0.97 (0.91, 1.03)	0.303
	rs7927129	0.05	0.98 (0.93, 1.02)	0.319	0.99 (0.95, 1.03)	0.655
	rs11023831	0.02	0.96 (0.89, 1.03)	0.275	0.99 (0.93, 1.06)	0.759
	rs462402	0.38	1.06 (1.02, 1.09)	0.001	1.04 (1.01, 1.07)	0.013
	rs463924	0.89	0.95 (0.92, 0.99)	0.014	0.97 (0.93, 1.00)	0.035
	rs231841	0.77	0.95 (0.91, 0.98)	0.002	0.97 (0.94, 1.00)	0.025
	rs231840	–	0.95 (0.91, 0.98)	0.004	0.97 (0.94, 1.00)	0.076
	rs2283197	0.79	0.95 (0.92, 0.99)	0.011	0.97 (0.94, 1.00)	0.086
	rs2283200	0.03	1.03 (0.95, 1.10)	0.502	1.02 (0.95, 1.08)	0.618
	rs231901	0.02	0.96 (0.91, 1.01)	0.154	0.98 (0.93, 1.03)	0.369
	rs4930149	0.03	0.98 (0.92, 1.04)	0.506	0.99 (0.94, 1.04)	0.616
	rs2237892	0.00	0.95 (0.89, 1.02)	0.147	0.94 (0.89, 0.99)	0.031

Adiponectin (μg/ml)	rs2334499	0.00	1.00 (0.97, 1.04)	0.818	1.01 (0.98, 1.04)	0.685
	rs3817198	0.00	1.00 (0.96, 1.04)	0.887	1.00 (0.96, 1.03)	0.784
	rs231362	0.18	0.97 (0.94, 1.01)	0.129	0.97 (0.94, 1.00)	0.064
	rs7105073	0.02	0.96 (0.89, 1.03)	0.265	0.99 (0.93, 1.05)	0.685
	rs7128926	0.06	0.97 (0.93, 1.02)	0.258	0.99 (0.95, 1.03)	0.578
	rs7941377	0.03	0.96 (0.89, 0.04)	0.302	0.97 (0.91, 1.04)	0.410
	rs231357	0.41	0.95 (0.92, 0.99)	0.007	0.96 (0.93, 0.98)	0.002
	rs11023793	0.05	0.99 (0.95, 1.04)	0.721	1.00 (0.96, 1.04)	0.976
	rs16928561	0.02	0.93 (0.86, 1.00)	0.055	0.98 (0.92, 1.04)	0.502
	rs7927129	0.05	0.97 (0.92, 1.01)	0.170	0.99 (0.95, 1.03)	0.755
	rs11023831	0.02	0.95 (0.88, 1.03)	0.209	0.97 (0.91, 1.04)	0.396
	rs462402	0.38	1.04 (1.00, 1.08)	0.033	1.04 (1.01, 1.07)	0.017
	rs463924	0.89	0.98 (0.94, 1.02)	0.363	0.97 (0.94, 1.00)	0.070
	rs231841	0.77	0.98 (0.95, 1.02)	0.379	0.98 (0.95, 1.01)	0.164
	rs231840	–	0.97 (0.93, 1.01)	0.175	0.97 (0.94, 1.01)	0.096
	rs2283197	0.79	0.97 (0.94, 1.01)	0.130	0.97 (0.94, 1.00)	0.076
	rs2283200	0.03	0.95 (0.88, 1.02)	0.186	0.96 (0.90, 1.02)	0.146
	rs231901	0.02	0.96 (0.91, 1.02)	0.178	0.99 (0.94, 1.03)	0.540
	rs4930149	0.03	0.98 (0.93, 1.05)	0.603	0.99 (0.94, 1.05)	0.816
	rs2237892	0.00	0.98 (0.92, 1.05)	0.621	0.98 (0.92, 1.04)	0.448

Regression analysis of neighbouring SNP genotypes predicting metabolic measures M and adiponectin for the genotyped RISC cohort (n = 1119). Table shows unadjusted analyses and adjusted for confounders. Regression analysis outcomes are presented as: (exponent of regression coefficient (95% confidence interval), p-value). ffm, fat-free mass.

SNP rs231357 was correlated with methylation at rs231840 (0.64 (0.61, 0.68), p = 1.786e−48). SNP rs231357 was also associated with methylation at the local CpG sites adjacent to rs231840 adjusted for confounders (1.00 (0.99, 1.00), p = 0.013). However, the direct effect of genotype indicated that only 1.32% of the variance in methylation at rs231840 was attributable to rs231357 genotype, compared to 6.01% variance attributable to rs231840 genotype. The association between rs231357 and methylation at rs231840 remained following Bonferroni correction. However, the association between this SNP and average methylation at the local CpG sites did not remain after correction.

SNP rs231357 has a minor allele frequency of 0.48 in the RISC population; presence of the minor allele created a CpG site that had the potential to become methylated. SNPs rs231840 and rs231357 were not in LD with any of the SNPs in this region previously associated with type 2 diabetes (rs2334499, rs231362, rs2237892), with the exception of rs231362 which was in weak LD (see Fig. 1). We did not observe a strong association between rs231362 and either M or adiponectin in this study.

### 3.7 DNA methylation in *KCNQ1* is correlated between tissues using publically available data

**Fig. 4:**
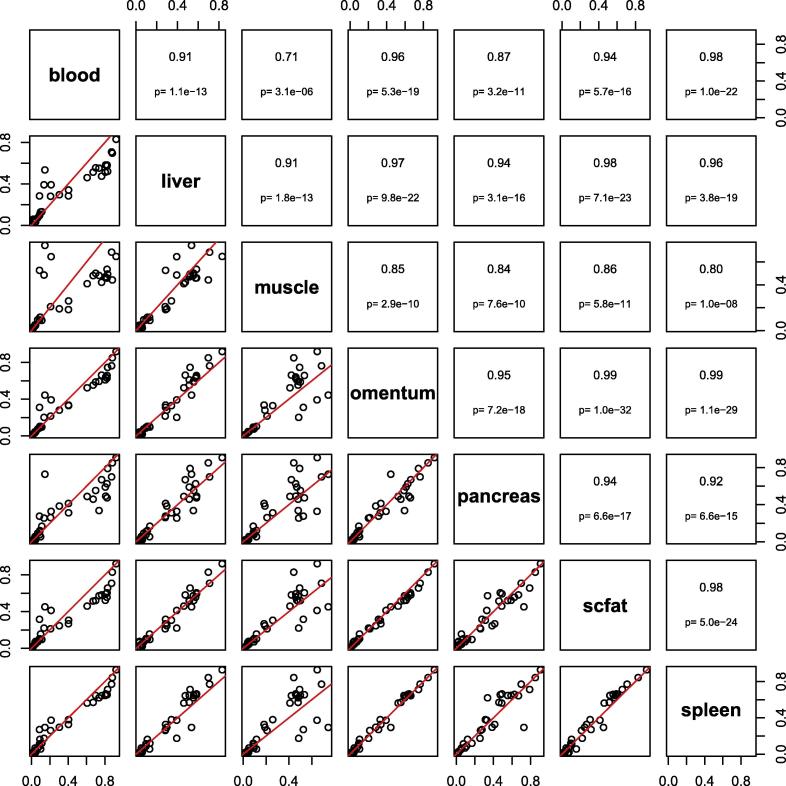
Pairwise comparisons of *KCNQ1* CpGs between blood and other tissues using publicly available array data. Six tissue types are shown (blood: n = 11; muscle, omentum, and subcutaneous fat: n = 6; liver: n = 5; pancreas: n = 4). The upper panel shows the Pearson correlation coefficient and P values; the lower panel shows the pairwise scatterplot (trend line shown in red). Data are a subset of Gene Expression Omnibus data entry GSE48472 [20]. (For interpretation of the references to colour in this figure legend, the reader is referred to the web version of this article.)

Since DNA methylation in this study was measured in blood we sought to identify how methylation measured within the *KCNQ1* gene varied between tissue types. We evaluated all CpG sites annotated to *KCNQ1* in subset of data from the Gene Expression Omnibus data entry GSE48472. This dataset included data from blood and a range of other tissues, including pancreas, fat, and muscle [20]. Although sample numbers were small, mean methylation in blood versus other tissues showed high levels of correlation with Pearson correlation coefficients of between 0.71 and 0.98 (see Fig. 4). This data suggests that measurement of DNA methylation in blood may be representative of other tissues at this locus.

## 4 Discussion

Investigation of DNA methylation at the *KCNQ1* locus demonstrated an association between methylation levels and insulin sensitivity in a healthy cohort. Differential methylation at rs231840 was driven by a polymorphic substitution of a cytosine residue within a CpG site (rs231840), creating an obligatory change in methylation. This obligatory alteration appeared to have a localised influence on surrounding methylation of CpG sites whose methylation levels were correspondingly lowered in the presence of the variant allele at rs231840. It is unknown whether the mechanism for this observation is SNP dependent or due to effects of co-methylation whereby reduced methylation of CpG site at rs231840 affects nearby methylation rather than as an effect of the SNP itself.

We did not observe an association between DNA methylation and insulin sensitivity at rs231840 when restricting analyses to C:C carriers. However, it is difficult to determine the relative importance of genotype and methylation status amongst this subgroup as the sample size in this analysis was small and the variance in DNA methylation was also low (see Fig. 2).

It was noted that CpG 5 (Fig. 1) was the CpG site most strongly influenced by rs231840 genotype. This may have occurred because it is the CpG site closest to rs231840, or because rs231840 influences methylation preferentially in the upstream direction.

It was observed that alleles contributing to reduced insulin sensitivity and adiponectin levels also contributed to reduced methylation at rs231840 and adjacent CpG sites. Methylation may indicate one mechanism whereby sequence variation influences a type 2 diabetes-related trait, i.e. the SNP influences the epigenetic regulation of expression, or tags a variant with a functional effect. One further potential explanation may be pleiotropic influences of rs231840 which may act through both epigenetic regulatory pathways and through its influence on protein function. Although publicly available data indicated methylation measured in blood was similar in other tissue types we were not able to directly measure this in our cohort at the CpG sites reported in this study.

Exploration of local genetic variation identified a second SNP, rs231357, which was associated with both methylation and insulin sensitivity. The association between genotype and metabolic measure was stronger than the effect seen for rs231840. This suggests rs231357 could either be causal, or be more closely tagging a causal variant than rs231840. SNP rs231357 was also associated with methylation surrounding rs231840. Both rs231840 and rs231357 alter the possibility of methylation occurring when the minor allele is present; rs231840 ablates a CpG site, and rs231357 introduces one. Further study investigating these SNPs as haplotypes is warranted.

The influence of site specific methylation on insulin sensitivity and adiponectin in this study is very modest (<1%). However, this is compatible with single SNP associations with traits such as height, where multiple SNPs of small effect can act to collectively exert a substantial difference on phenotype [21]. This scenario may also apply in the context of DNA methylation variation.

### 4.1 Potential mechanisms of action

In this study, differential methylation is partly determined by genotype, representing a primary genetic driver of epigenetic variation. However, it is inconclusive from these data whether rs231840 and rs231357 are exerting their influence via methylation mediated changes in gene expression or through another mechanism. A recent study suggested that methylation was on the causal pathway between genetic variation in *KCNQ1* and type 2 diabetes in later life [22] which supports the former hypothesis. A further study has shown that genotype influences both methylation and gene expression throughout the genome [23]. It is thought that the frequency at which SNPs affect local methylation is somewhere between 4.0% and 8.6% [24], [25]. Although relatively uncommon, it is plausible that certain haplotypes may lead to altered methylation profiles due to their underlying genetic architecture.

### 4.2 Potential role of *KCNQ1* mediating insulin sensitivity and beta cell function

Common variation in *KCNQ1* has been shown to increase susceptibility to type 2 diabetes across populations of different ethnic backgrounds [1], [11]. The evidence to date suggests that this is mediated via altered insulin secretion [26], [27] although this was not confirmed in the most recent type 2 diabetes GWAS where the postulated mechanism of action for *KCNQ1* was designated unclassified [1]. In this study we identify an association between variation in *KCNQ1* and whole-body insulin sensitivity determined using the gold-standard euglycaemic-hyperinsulinaemic clamp. This association between methylation levels and insulin sensitivity does not overlap with those variants at the *KCNQ1* locus previously linked to type 2 diabetes risk via insulin secretion impairment. Although genetic association studies have primarily identified associations between SNPs and insulin secretion defects, more recently a number of these genes have also been associated with insulin sensitivity including PPARG, KLF14, IRS1 and GCKR [28].

The majority of studies linking *KCNQ1* with insulin secretion defects and type 2 diabetes risk have focused upon patients with type 2 diabetes. Two previous studies have examined *KCNQ1* variants (rs2283228 and rs2237895) in individuals without diabetes but found no relationship with an OGTT derived measure (Matsuda ISI) of insulin sensitivity [26], [29]. HapMap data suggests that genotypes of these variants are not correlated with rs231840 or rs231357. However, decreased *KCNQ1* expression has been reported in a knockout mouse model leading to increased whole body insulin sensitivity and increased insulin mediated glucose uptake in liver and muscle [30]. The mechanisms by which altered *KCNQ1* expression impacted upon insulin action were not defined, but these observations are in keeping with our findings that variation in *KCNQ1* is linked to whole body insulin sensitivity.

Our data shows a lack of corroboration of whole-body insulin sensitivity measures (clamp) and fasting-based measures of insulin resistance (HOMA-IR, fasting glucose and insulin measures) with regard to association with genotype or methylation. However, HOMA-IR and insulin sensitivity ascertained by euglycaemic-hyperinsulinaemic clamp are only moderately genetically correlated (r = −0.53), in keeping with differing aetiological pathways [31].

### 4.3 Limitations

One of the main limitations of this study was the inability to investigate parent-of-origin effects in our cohort. This is of interest, since *KCNQ1* is an imprinted gene and the region of *KCNQ1* investigated lies within an imprinting control region, responsible for regulating parent-of-origin specific expression of a number of genes in the region [32]. Additionally, upstream of the *KCNQ1* region examined in this paper is the noncoding RNA *KCNQ1* overlapping transcript 1 (*KCNQ1OT1).* The possible role of *KCNQ1OT1* expression in the pathogenesis of type 2 diabetes and its parent-of-origin effect has been reported in mice [33]. This avenue could be further explored in a second cohort, where parental samples are available.

We were also unable to assess the effect of rs231840 on mRNA-quantified expression data in this cohort, since no expression data has been collected. The largest publicly available dataset reporting eQTMs (expression quantitative trait methylation) we could identify reports methylation associations with gene expression in *cis* using approximately 2000 whole blood samples [34]. From their results several CpG sites in the *KCNQ1* gene region appear to correlate with expression of other genes in *cis* (including *TSPAN32*, *C11orf21*, *CD81* and *CDKN1C*) and it appears that the network of associations between gene expression and methylation is complex. For *KCNQ1* there is the additional complicating factor that methylation-expression relationships may have a parent-of-origin effect which we are unable to address either using our data or that of publicly available sources.

Whole blood contains a variety of cell types and consequently differences in cellular composition could confound analyses conducted on heterogeneous sample types. Although we do not predict that cell composition would vary by metabolic status we were unable to address this possibility since cell counts were not measured and could not be computationally estimated within our dataset.

A further potential limitation is that of the relatively young age of the RISC cohort since it is possible that the metabolic measures obtained may not be an accurate reflection of the genetic risk. Finally, although sample size was as large as possible, some statistical tests may have had limited power.

### 4.4 Conclusions

This important work highlights an emerging field of analysis, investigating the association between CpG methylation (known to influence gene expression) and underlying genetic architecture.

We report an association between *KCNQ1* SNP rs231357 and insulin sensitivity. This SNP is not in substantial LD with any *KCNQ1* SNPs previously associated with type 2 diabetes or any other diabetes related metabolic measures.

Further work is needed to confirm the association between *KCNQ1* SNPs and insulin sensitivity, and to test any association between genotype and overt type 2 diabetes in other study populations. We hypothesise that methylation may play a role in the mechanism of action of these genetic alterations and there may be a parent-of-origin dependent effect.

## 5 Authors’ contributions

UJS did data generation, data analysis and revised the manuscript. WX did imputation of genotypes and reviewed earlier manuscript versions. AF participated in study design, analyzed adiponectin and reviewed manuscript. JJN contributed to the design, recruitment and conduct of the clinical and physiological studies. KH contributed to the conception and design of the main RISC study, recruitment and examination of the study participants, interpretation of the results and review of manuscript. MW and CLR designed study, interpreted data and reviewed manuscript. HRE designed study, analyzed data, interpreted data and wrote the manuscript. Detailed phenotyping of RISC cohort was obtained from RISC consortium.

## References

[1] Scott R.A., Scott L.J., Mägi R., Marullo L., Gaulton K.J., Kaakinen M. (2017). An expanded genome-wide association study of type 2 diabetes in Europeans. Diabetes.

[2] Voight B.F., Scott L.J., Steinthorsdottir V., Morris A.P., Dina C., Welch R.P. (2010). Twelve type 2 diabetes susceptibility loci identified through large-scale association analysis. Nat Genet.

[3] Davegårdh C., García-Calzón S., Bacos K., Ling C. (2018). DNA methylation in the pathogenesis of type 2 diabetes in humans. Mol Metab.

[4] Walaszczyk E., Luijten M., Spijkerman A.M.W., Bonder M.J., Lutgers H.L., Snieder H. (2017). DNA methylation markers associated with type 2 diabetes, fasting glucose and HbA_1c_ levels: a systematic review and replication in a case–control sample of the lifelines study. Diabetologia.

[5] Nilsson E., Jansson P.A., Perfilyev A., Volkov P., Pedersen M., Svensson M.K. (2014). Altered DNA methylation and differential expression of genes influencing metabolism and inflammation in adipose tissue from subjects with type 2 diabetes. Diabetes.

[6] Dayeh T., Volkov P., Salö S., Hall E., Nilsson E., Olsson A.H. (2014;10.). Genome-wide DNA methylation analysis of human pancreatic islets from Type 2 diabetic and non-diabetic donors identifies candidate genes that influence insulin secretion. PLoS Genet.

[7] Bell C.G., Finer S., Lindgren C.M., Wilson G.A., Rakyan V.K., Teschendorff A.E. (2010;5.). Integrated genetic and epigenetic analysis identifies haplotype-specific methylation in the FTO type 2 diabetes and obesity susceptibility locus. PLoS One.

[8] Chambers J.C., Loh M., Lehne B., Drong A., Kriebel J., Motta V. (2015). Epigenome-wide association of DNA methylation markers in peripheral blood from Indian Asians and Europeans with incident type 2 diabetes: a nested case-control study. Lancet Diabetes Endocrinol.

[9] Arner P., Sahlqvist A.S., Sinha I., Xu H., Yao X., Waterworth D. (2016). The epigenetic signature of systemic insulin resistance in obese women. Diabetologia.

[10] Hidalgo B., Irvin M.R., Sha J., Zhi D., Aslibekyan S., Absher D. (2014). Epigenome-wide association study of fasting measures of glucose, insulin, and homa-ir in the genetics of lipid lowering drugs and diet network study. Diabetes.

[11] Sun Q., Song K., Shen X., Cai Y. (2012). The association between KCNQ1 gene polymorphism and type 2 diabetes risk: a meta-analysis. PLoS One.

[12] Kong A., Steinthorsdottir V., Masson G., Thorleifsson G., Sulem P., Besenbacher S. (2009). Parental origin of sequence variants associated with complex diseases. Nature.

[13] Beatty L., Weksberg R., Sadowski P.D. (2006). Detailed analysis of the methylation patterns of the KvDMR1 imprinting control region of human chromosome 11. Genomics.

[14] Hills S.A., Balkau B., Coppack S.W., Dekker J.M., Mari A., Natali A. (2004). The EGIR-RISC Study (the European group for the study of insulin resistance: Relationship between insulin sensitivity and cardiovascular disease risk): I. Methodol. Object. Diabetologia.

[15] Pascoe L., Tura A., Patel S.K., Ibrahim I.M., Ferrannini E., Zeggini E. (2007). Common variants of the novel type 2 diabetes genes CDKAL1 and HHEX/IDE are associated with decreased pancreatic β-cell function. Diabetes.

[16] Pascoe L., Frayling T.M., Weedon M.N., Mari A., Tura A., Ferrannini E. (2008). Beta cell glucose sensitivity is decreased by 39% in non-diabetic individuals carrying multiple diabetes-risk alleles compared with those with no risk alleles. Diabetologia.

[17] Finucane F.M., Luan J., Wareham N.J., Sharp S.J., O’Rahilly S., Balkau B. (2009). Correlation of the leptin: adiponectin ratio with measures of insulin resistance in non-diabetic individuals. Diabetologia.

[18] Takeuchi F., Serizawa M., Yamamoto K., Fujisawa T., Nakashima E., Ohnaka K. (2009). Confirmation of multiple risk loci and genetic impacts by a genome-wide association study of type 2 diabetes in the Japanese population. Diabetes.

[19] Unoki H., Takahashi A., Kawaguchi T., Hara K., Horikoshi M., Andersen G. (2008). SNPs in KCNQ1 are associated with susceptibility to type 2 diabetes in East Asian and European populations. Nat Genet.

[20] Slieker R.C., Bos S.D., Goeman J.J., Bovee J.V., Talens R.P., van der Breggen R. (2013). Identification and systematic annotation of tissue-specific differentially methylated regions using the Illumina 450k array. Epigenetics Chromatin.

[21] Lango Allen H., Estrada K., Lettre G., Berndt S.I., Weedon M.N., Rivadeneira F. (2010). Hundreds of variants clustered in genomic loci and biological pathways affect human height. Nature.

[22] Elliott H.R., Shihab H.A., Lockett G.A., Holloway J.W., McRae A.F., Smith G.D. (2017). Role of DNA methylation in type 2 diabetes etiology: using genotype as a causal anchor. Diabetes.

[23] Bell J.T., Pai A.A., Pickrell J.K., Gaffney D.J., Pique-Regi R., Degner J.F. (2011;12.). DNA methylation patterns associate with genetic and gene expression variation in HapMap cell lines. Genome Biol.

[24] Gibbs J.R., van der Brug M.P., Hernandez D.G., Traynor B.J., Nalls M.A., Lai S.L. (2010). Abundant quantitative trait loci exist for DNA methylation and gene expression in Human Brain. PLoS Genet.

[25] Zhang D., Cheng L., Badner J.A., Chen C., Chen Q., Luo W. (2010). Genetic control of individual differences in gene-specific methylation in human brain. Am J Hum Genet.

[26] Jonsson A., Isomaa B., Tuomi T., Taneera J., Salehi A., Nilsson P. (2009). A variant in the KCNQ1 gene predicts future type 2 diabetes and mediates impaired insulin secretion. Diabetes.

[27] Müssig K., Staiger H., MacHicao F., Häring H.U., Fritsche A. (2010). Genetic variants affecting incretin sensitivity and incretin secretion. Diabetologia.

[28] Dimas A.S., Lagou V., Barker A., Knowles J.W., Mägi R., Hivert M.-F. (2014). Impact of type 2 diabetes susceptibility variants on quantitative glycemic traits reveals mechanistic heterogeneity. Diabetes.

[29] Stančáková A., Kuulasmaa T., Paananen J., Jackson A.U., Bonnycastle L.L., Collins F.S. (2009). Association of 18 confirmed susceptibility loci for type 2 diabetes with indices of insulin release, proinsulin conversion, and insulin sensitivity in 5327 nondiabetic Finnish men. Diabetes.

[30] Boini K.M., Graf D., Hennige A.M., Koka S., Kempe D.S., Wang K. (2009). Enhanced insulin sensitivity of gene-targeted mice lacking functional KCNQ1. AJP Regul Integr Comp Physiol.

[31] Holzinger U., Kitzberger R., Fuhrmann V., Funk G.C., Madl C., Ratheiser K. (2007). Correlation of calculated indices of insulin resistance (QUICKI and HOMA) with the euglycaemic hyperinsulinaemic clamp technique for evaluating insulin resistance in critically ill patients. Eur J Anaesthesiol.

[32] Thakur N., Tiwari V.K., Thomassin H., Pandey R.R., Kanduri M., Göndör A. (2004). An antisense RNA regulates the bidirectional silencing property of the Kcnq1 imprinting control region. Mol Cell Biol.

[33] Asahara S., Etoh H., Inoue H., Teruyama K., Shibutani Y., Ihara Y. (2015). Paternal allelic mutation at the Kcnq1 locus reduces pancreatic β-cell mass by epigenetic modification of Cdkn1c. Proc Natl Acad Sci USA.

[34] Bonder M.J., Luijk R., Zhernakova D.V., Moed M., Deelen P., Vermaat M. (2017). Disease variants alter transcription factor levels and methylation of their binding sites. Nat Genet.

